# A New Instrument for Cauterizing the Urethra

**Published:** 1852-07

**Authors:** E. S. Cooper

**Affiliations:** Peoria, (Ill.)


*A new instrument for cauterizing the Urethra.* By E. S. Cooper, M. D. Reported by L. C. Lane, M. D., of Peoria, Ill.

An instrument for cauterizing the urethra has been invented by Dr. Cooper of this place, which for facility of application and certainty of results is superior to all other means hitherto used, combined.

It consists of a copper catheter, with the end for half an inch a little smaller than the body, and perforated with several holes. This is introduced down to the stricture, and then filled with dilute nitric acid, which, acting on the copper, soon produces the nitrate, which coming in contact with the urethra through the holes, produces cauterization to the extent desired.

The strength of the solution and the length of time the instrument is permitted to remain, regulates the degree of cauterization completely. Dr. Cooper generally uses one third of nitric acid, and two of water, and permits the instrument to remain for one and a half minutes, though a much shorter time will often answer.

The shape of the instrument may be varied to suit the case; thus, when several strictures exist in the strait part of the urethra a a strait catheter might be used, with holes at several places to correspond to their number and location.

Though great contrariety of opinion exists among medical men in regard to the degree of cauterization most valuable, this instrument commends itself alike to all; for whether it is believed that caustics should be applied boldly so as to cause the detachment of a slough, and thus physically enlarge the canal, or by a slighter application modify the action of the lining, the variations are easily made with it.

*Peoria, (Ill.) April 15th,* 1852.

